# Genome-driven insights into *Bacillus safensis* strain B7 as a seed coating agent for plant growth promotion and alleviation of biotic and abiotic stresses

**DOI:** 10.1371/journal.pone.0329619

**Published:** 2025-08-18

**Authors:** Maissa Ben-Jabeur, Zayneb Kthiri, Salma Jallouli, Kalthoum Harbaoui, Zoubeir Chamekh, Sawsen Ayadi, Youssef Trifa, Walid Hamada

**Affiliations:** 1 Carthage University, Laboratory of Genetics and Cereal Breeding (LR14 AGR01), The National Agronomic Institute of Tunisia, Tunis, Tunisia; 2 Carthage University, Higher Institute of Preparatory Studies in Biology and Geology (ISEP BG), Soukra, Tunisia; 3 Carthage University, Higher School of Agriculture of Mateur, Mateur, Tunisia; 4 Carthage University, National Institute of Agronomic research of Tunis (INRAT), Tunis, Tunisia; Graphic Era Institute of Technology: Graphic Era Deemed to be University, INDIA

## Abstract

Despite the significant increase in the use of plant growth-promoting bacteria (PGPB) in agriculture, there is a dearth of studies addressing the impact of seed coating with PGPB on plant productivity. The main objective of this research is to evaluate the potential of *Bacillus safensis* strain B7 as a seed coating agent to confer plant growth promotion and tolerance against biotic and abiotic stress. The whole-genome sequencing of strain B7 was also performed to study its genomic features. The effect of seed coating with strain B7 was assessed on seed water uptake, germination and seedling dry weight under controlled conditions. Besides, the direct and indirect effect against biotic stress was evaluated through antifungal activity and potential in stimulating the induced systemic resistance (ISR) under controlled conditions. Afterwards, the effect was evaluated in the field under drought stress conditions, based on the traits of grain yield (GY), straw yield, number of spikes/m^2^, and thousand kernel weight (TKW). It is noticeable that seed coating with strain B7 resulted in greater and faster water uptake. Germination and plant growth similarly boosted. Strain B7 was able to hamper the mycelial growth of *F. culmorum* and *S*. *sclerotiorum* and to induce ISR, after *B. cinerea* infection, in melon leaves taken from root-treated plants. In the field, seed coating with strain B7 improved wheat performance under the different environments and mitigated the effect of drought on spikes/m^2^, GY and TKW. The observed impact on wheat plants is supported by genomic analysis of strain B7 showing the presence of genes related to beneficial plant–bacteria interactions involved in plant colonization, growth promotion and alleviation of stress. Therefore, *B. safensis* strain B7 has promising applications in the seed treatment industry to increase plant yield and alleviate the impact of stress.

## Introduction

Global climate change, particularly increased heat and drought stress, threatens food security by directly reducing crop yields. To address these challenges, various strategies have been developed, including omics-based breeding, integrated water use technologies, and sustainable agricultural practices [1]. However, some of these approaches are economically unviable, cost-intensive, and time-consuming. In parallel, the excessive use of chemical fertilizers contributes to environmental pollution and degradation [2,3]. These limitations drive the search for agronomic strategies that are eco-friendly, simple, efficient, sustainable, and capable of improving productivity.

Microbial-based biostimulants, particularly plant growth-promoting bacteria (PGPB), have emerged as promising tools in sustainable agriculture thanks to their dual role in enhancing plant development and stress resilience [4,5]. Among various delivery methods, seed coating technology offers a targeted and resource-efficient approach. Seed coating with PGPB ensures early root–microbe interactions that stimulate germination, enhance seedling vigour, and provide protection against biotic and abiotic stresses [6,7].

Several studies have shown that chemical or biological binders, fillers, polymers, and colorants are essential components of seed coating formulations [6,8,9]. However, traditional chemical-based seed coatings raise environmental concerns due to the non-biodegradable carriers and synthetic dyes, which contribute to pollution and handling risks. In response, regulatory frameworks are evolving, and modern seed coating technologies are being designed to minimize off-target ecological impacts [8,10]. Natural alternatives are promising as they are biodegradable, non-toxic, and improve adhesion and uniformity in coatings [9]. The selection of coating materials is therefore critical, not only for ensuring seed germination and plant development but also for maintaining the viability of microbial inoculants and minimizing environmental impacts [6]. Furthermore, without an appropriate carrier, seed coating with PGPB often fails to support effective crop performance under field conditions, despite showing promise under controlled conditions [11]. These challenges highlight the need to evaluate both coating materials and microbial inoculants within seed coating systems, especially under field conditions. In this context, our interest was drawn to tannin-based seed coating as a natural, eco-friendly carrier for the PGPB inoculum.

*Bacillus* species are among the most extensively studied PGPB, owing to their robust stress tolerance, spore-forming capacity, and diverse plant-beneficial traits [12]. The isolation of PGPB from extreme environments has proven effective in identifying strains with enhanced resilience and plant-colonizing ability. In this context, we recently isolated *B. safensis* strain B7 from surface-sterilized leaves of *Diplotaxis harra*, a native plant adapted to arid conditions of heat, drought, and salinity. This species exhibits strong potential as a microbial inoculant owing to its ability to colonize a wide range of habitats, promote plant growth, and alleviate the impact of biotic and abiotic stresses [13–18].

Despite increasing interest in *B. safensis*, its application in a seed coating system—particularly for cereals like wheat under drought stress, remains unexplored. Furthermore, few studies have integrated genomic analysis to validate and explain the mechanisms underlying observed field-level benefits [19]. Therefore, the objectives of the study are: (i) to evaluate the impact of seed coating with strain B7 on seed germination, plant growth, and wheat performance under drought conditions in the field; and (ii) to analyse the genome of strain B7 to identify genes involved in plant colonization, growth promotion, and stress mitigation. To our knowledge, this is the first study to combine genomic insights with agronomic evaluation of *B. safensis* as a seed coating agent in wheat.

## Materials and methods

### Isolation and genotyping of *Bacillus safensis* strain B7

#### Isolation and genome sequencing.

*B. safensis* strain B7 was isolated from the leaves of *Diplotaxis harra*, which was collected from the arid region of Tunisia (Bou Ghrara, Mednine). The strain was isolated after surface sterilization of the leaves by a stepwise washing procedure. The genomic DNA of strain B7 was subjected to whole-genome sequencing using the Illumina NovaSeq 6000. The isolation procedure, full genomic features, and phylogenetic analysis results have been made available in Zenodo at https://zenodo.org/records/10569252 [20]. Sequence and genotype data are publicly available at the corresponding NCBI databases: whole-genome shotgun (WGS) and short read archive (SRA) (BioProject PRJNA1011052).

#### Genome-based taxonomic classification.

For taxonomic assignment, the Type (Strain) Genome Server (TYGS) [21] was employed. To reliably identify strain B7, the genomes of the resulting type strains were used to calculate the values of average nucleotide sequence identity (ANI) and digital DNA–DNA hybridization (dDDH) between the genome of strain B7 and its closely related strains. ANI was calculated using the FastANI tool via the Proksee server [22] (https://proksee.ca/), while digital DNA–DNA hybridization (dDDH) values were computed using the Genome-to-Genome Distance Calculator provided by DSMZ (GGDC 3.0; https://ggdc.dsmz.de/ggdc.php#), applying formula 2, which is the recommended formula for draft genomes [23].

A phylogenetic tree was constructed using whole genome sequence datasets of *B. safensis* strain B7 and the reference type strains via the Reference Sequence Alignment Based Phylogeny Builder (REALPHY) online pipeline (https://realphy.unibas.ch/realphy/) [24]. The resulting tree was visualized using the Interactive Tree Of Life (iTOL) version 7 online tool (https://itol.embl.de/) [25].

#### Gene prediction and annotations.

The genome was annotated using the NCBI Prokaryotic Genome Annotation Pipeline (PGAP) and PROKKA. For consistency, the genomes of *strain B7* and its closely related strains were re-annotated using the Rapid Annotation using Subsystem Technology (RAST) pipeline [26]. Annotation was also performed using the Pathosystems Resource Integration Center (PATRIC) via the BV-BRC server (https://www.bv-brc.org/), where the antibiotic resistance genes were identified [27]. To highlight genes involved in plant growth-promoting activity, functional annotation was additionally carried out using the web-based RAST annotation option available in Proksee server.

#### Genome comparison analysis.

The Multi Genome Compare tool of the SEED viewer was used for genome comparison, with *strain B7* as the reference. It was compared to four other *B. safensis* strains: GX-H6, H31R-08, PgKB20, and PLA 1006 [28]. This system performs a bidirectional BLAST comparison between each genome and the reference. The results are categorized as either “bi” (bidirectional best hit) or “uni” (unidirectional best hit). A threshold of 98% sequence identity was applied to identify highly similar genomes.

In addition, a pan- and core-genome analysis was conducted using the Integrated Prokaryotes Genome and pan-genome Analysis (IPGA) v1.09 online tool (https://nmdc.cn/ipga) [29]. This analysis included the genome sequences of *B. safensis* strain B7 and the same four comparative strains. The core genome represents genes shared by all strains, and the pan-genome encompass both core and accessory genes.

#### Genome mining for secondary metabolite synthesis gene clusters.

The genome sequence of B7 strain was analysed for secondary metabolite biosynthetic gene clusters using the bioinformatics tools ‘antibiotics and Secondary Metabolite Analysis Shell’ (antiSMASH) (v. 7.0; https://antismash.secondarymetabolites.org; relaxed setting) [30], ‘Natural Product Domain Seeker’ (NaPDoS2) (https://npdomainseeker.sdsc.edu/) [31], and the bacteriocin-specific software ‘BActeriocin GEnome Mining TooL’ (BAGEL 4) (http://bagel4.molgenrug.nl/) [32]. In addition, a BLAST search was performed on the predicted clusters to identify the closest homologues in the available database.

### Plant material and seed coating treatment

In this study, the Tunisian cultivar Karim of durum wheat (*Triticum turgidum*) —obtained from the National Gene Bank of Tunisia —was used. The coating technique consisted of preparing, for each 10 g of wheat seeds, a mixture containing 40 μl of the coating agent Agritan® SC (Silvateam, San Michele di Mondovì, Italy) and 400 μl of either the bacterial inoculum (10^6^ CFU.ml^-1^) or water (used as the coated control, CC). The coating mixture was then applied progressively to wheat seeds in a continuous rotation until complete adhesion and absorption, following the method of Ben-Jabeur et al. [33]. The experiment also included a non-coated control (NCC).

The coating agent Agritan® SC was used to replace the chemical coating agent Agicote Rouge T17, which we used in previous studies [34]. Agritan® SC is not an inert coating agent; it is a water-based formulation composed of tannin extract and a reddish-brown liquid used as a natural resin to encrust alfalfa seeds. It is an environmentally friendly, organic product that forms a homogeneous and adhesive film on the seeds. In our study, to achieve an inert effect, the appropriate dose (9.09%, corresponding to 40 µL of Agritan® SC added to 400 µL of water or treatment solution) was selected based preliminary trials. These trials involved applying different doses of Agritan® SC as wheat seed coating treatments with the goal of achieving optimal adhesion, without promoting germination or seedling growth, and without stimulating phenolic content or peroxidase activity during germination, as shown in our previous research [34]. The tested doses were 40 µL, 50 µL, 60 µL µL, and 70 µL added to 400 µL of water or treatment solution, corresponding to the doses of 0.09%, 11.1%, 13.04%, 14.89%, respectively. The effect of the different Agritan® SC doses on seed germination is provided in the supplementary data (S1 Fig, S0 File).

### *In vitro* experiment: The impact of seed coating on seed germination, water uptake, and seedling development in wheat

#### Seed germination.

To investigate early germination, germinating wheat seeds were counted at 3 days post coating (dpc), and the percentage of seed germination was calculated using the following formula:


$$Seed\;germination\;(\%)=\left(\frac{Germinated\;seeds}{Total\;Seed}\right)\times \;100$$

#### Seed water uptake.

For each coating treatment, 3 replications of 9 seeds were taken from Petri dishes following the method of Tian et al. [35]. Seeds were weighed at time point zero (immediately after coating) and then every 5 h until the seed weight was stabilized, indicating saturation. Water uptake was calculated as follows:


$$\begin{array}{c} Water\;uptake\;(\%)=\;\left[\frac{\left(SWt\;-\;SW0\right)}{SW0}\right]\times \;100 \end{array}$$

Where SW0 is the Initial weight of seeds and SWt is the weight of seeds after absorbing water at a specific time point.

#### Shoot and root dry weight.

The dry weights of roots and shoots were measured at 10 dpc. Three plants per treatment were dried in an oven at 65 °C for 72 hours and then weighed.

### Biotic stress

#### *In vitro* assay for antifungal activity.

Two fungi were used in this assay. *Sclerotinia sclerotiorum* (SS01L.23) was isolated from infected lettuce plants in 2023, and *Fusarium culmorum* (FS1W) was isolated from infected durum wheat plants in 2022. The strains were cultured on Potato Dextrose Agar (PDA), and pure cultures were stored in the fungal collection of the crop science department of INAT, at 4°C. They were subcultured once a month.

The antifungal activity of strain B7 on *F. culmorum* and *S. sclerotiorum* was evaluated using a dual-culture plate assay. Two 5-mm diameter plugs of each pathogen were placed on opposite sides of a PDA Petri dish. A 3-cm-long streak of strain B7 was applied at the center, 2 cm from each fungal plug. PDA plates inoculated with pathogens alone served as controls. Each experiment was repeated three times and incubated at 20 °C until the pathogens completely colonized the control plates. To assess antifungal activity, the mean of radius of the fungal colonies in the test plates (T) and those in the control plates (C) was measured. The antifungal activity of the strain B7 was calculated as an inhibition percentage (I%) as follow:


$$\begin{array}{c} I\;(\%)=\;\left[\frac{\left(C\;-\;T\right)}{C}\right]\times \;100 \end{array}$$

#### Investigation of *B. safensis* potential in triggering systemic induced resistance (ISR) in melon plants through measurement of phenolic content and peroxidase activity in leaves and through detached leaves assay against *Botrytis cinerea.*

In this experiment, melon seedlings of the variety “Badii (DRM 3241)” obtained from Seminis (Bayer) were grown in a plant growth chamber. The impact of *B. safensis* B7 on seedling signalling was evaluated using a non-circulating hydroponics system, as described by Ben-Jabeur et al. [36]. After 10 days of growth, plants were treated at the root level by adding the bacterial inoculum to the nutritive solution. The bacterial inoculum was prepared in LB liquid medium at 25 °C for 48 h, and the bacterial concentration was adjusted to 10^9^ CFU.mL^−1^.

At 2-, 4-, and 6-days post-treatment (dpt), phenolic content and peroxidase activity were measured in the leaves. Peroxidases were extracted from 500 mg of leaf samples and measured calorimetrically at 470 nm using guaiacol as the hydrogen donor, following the method of Egley et al. [37]. Peroxidase activity was expressed as mmol.min^−1^.mg^−1^ P. Phenolic content was extracted from 500 mg of fresh leaves using the Folin–Ciocalteu method described by Singleton et al. [38]. Phenolic content was measured calorimetrically at 760 nm with catechol as the standard and expressed as mg. g^−1^ fresh weight (FW).

The ability of *B. safensis* B7 to trigger plant ISR against gray mold disease was also assessed using a detached leaf assay. At 3 dpt, leaves were excised and placed in Petri dishes containing moistened filter paper. Each leaf was inoculated on its lower surface with 6-mm-diameter mycelial plugs of *B. cinerea*, taken from the edge of 6-day-old PDA cultures. Petri dishes were covered and incubated at room temperature (25 °C) under a 12-hour photoperiod. Symptoms caused by *B. cinerea* were evaluated at 6 days post-inoculation (dpi), and the disease index was determined by measuring the average diameter of the necrotic area in both treated and untreated leaves.


$$Disease\;index\;(\%)=1-\left[\frac{\left(\;Leaf\;area\;-necrotic\;area\right)}{leaf\;area}\right]$$

### Abiotic stress: Drought

#### Field Experiment 1 in the semi-arid region of Tunisia.

The experiment was conducted during the 2019–2020 growing seasons at the experimental station of the Higher School of Agriculture of Mateur, located in northwestern Tunisia (37°03′ N, 9°36′ E). This region has a semi-arid climate. Weather conditions during the experimental season are presented in Table 1. The soil at the site is classified as a vertisol with a clay-silt texture based on its physical and chemical characteristics. A randomized complete block design with three replicates was used. Each plot consisted of 15 rows, each 1.5 m in length, with a row spacing of 0.5 m. Seedbed preparation followed a conventional method, involving medium-depth tillage followed by two passes with a disc harrow in early autumn. Sowing was carried out in the third week of December at a density of 350 seeds m^-2^.

**Table 1:** Climatic conditions during the 2019–2020 cropping season at the experimental station of the higher school of agriculture of mateur.

Month	December	January	February	March	April	May	June	SUM/Average
T min (°C)	8	7	7	8	8.5	10	18	9.5
T max (°C)	17	15	15.5	19	19.5	25	31	20.2
P (mm)	62	52	50	40	39	29	6	278

Abbreviations: P, precipitation; T, temperature.

#### Field Experiment 2 in the arid region of Tunisia.

The experiment was carried out during the 2016–2017 growing season at the experimental station in Chébika, located in central Tunisia (National Field Crops Institute, INGC, Kairouan, 35°37′ N, 9°56′ E). The region has an arid climate. Weather conditions during the experimental season are presented in Table 2. The soil at the site is characterized as a silty loam with slight salinity. A randomized complete block design with three replicates was used. Each plot consisted of 15 rows, each 1. 5 m in length, with a row spacing of 0.20 m. Sowing was carried out in the first week of December at a density of 300 seeds.m^-2^. All plots were drip-irrigated, and two supplemental irrigation (SI) levels were applied: 100% of SI (moderate drought stress) and 50% of SI (severe drought stress). These treatments were applied according to the method described by Ben-Jabeur et al. [33]. The frequency and duration of SI applications were adjusted based on several factors, including crop coefficient (Kc), reference evapotranspiration (ET_0_), and soil field capacity. Agronomic practices followed those commonly used at the INGC experimental station.

**Table 2:** Climatic conditions during the 2016–2017 cropping season at the experimental station in Chébika.

Month	December	January	February	March	April	May	June	SUM/Average
T min (°C)	11.3	6.3	9.1	10.8	10.8	17.3	21.3	12.4
T max (°C)	18.6	16.2	20.2	22.9	22.9	31.3	36.7	24.1
P (mm)	124.2	5.4	17.5	10.7	10.7	0.0	3.6	172.1
100% SI (mm)	0	130	120	115.7	96	0.0	0.0	461
Monthly available water under 100% SI (mm)	124.2	135.4	137.5	125	106.7	0	3.6	632.4
50% SI (mm)	0	60	60	50	45	0.0	0.0	300
Monthly available water under 50% SI (mm)	124.2	65.4	77.5	60.7	55.7	0	3.6	387.1

Abbreviations: P, precipitation; SI, supplemental irrigation; T, temperature.

#### Measurement of the agronomic components in both field experiments.

In each plot, a 1 m^2^ area in the central section was hand-harvested. The number of spikes per m^2^ was counted, and the straw yield was recorded. The number of grains per spike and the grain yield (GY, quintals/ha) were measured from the same 1 m^2^ harvested sample using a shredder (LD-180; Wintersteiger, Ried im Innkreis, Austria). The thousand kernel weight (TKW, g) was determined from a sample of 1,000 grains, counted using a Numigral X5 seed counter (CHOPIN Technologies, Villeneuve-la-Garenne, France).

#### Experimental sites access.

The field studies described in this investigation did not require special permits for site access or sample collection. Both experimental locations are not designated as private or protected areas. Access to the Higher School of Agriculture of Mateur was coordinated by co-author K Harbaoui, while access to the Chébika experimental station was arranged by co-author Y. Trifa.

#### Statistical analysis.

Under controlled conditions, a one-way analysis of variance (ANOVA) was used to evaluate the effect of treatments on seed germination, as well as on shoot and root dry weights. A two-way ANOVA (Treatment × HPC) was applied to assess the effect of treatments on pH and water uptake. Under field conditions, a one-way ANOVA was used to assess the effect of treatments on agronomic components in field experiment 1. For field experiment 2, a two-way ANOVA (Treatment × SI) was used to evaluate treatment effects on agronomic traits. Mean comparisons were performed using the least significant difference (LSD) test. All statistical analyses and graphical representations were conducted using RStudio software.

## Results and discussion

### Taxonomic classification based on phylogenetic analysis, average nucleotide identity, and digital DNA–DNA hybridization

**Fig 1:**
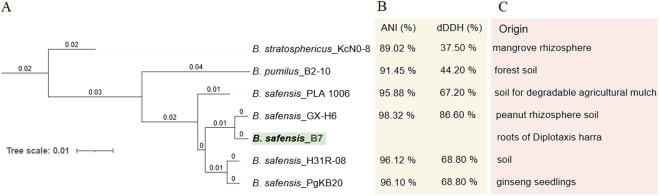
Taxonomic classification based on phylogenetic analysis, average nucleotide identity (ANI), and digital DNA–DNA hybridization (dDDH). (A) Whole-genome phylogenetic tree of *B. safensis* strains constructed with REALPHY and visualized with Itol. (B) dDDH and ANI values between *B. safensis* B7 and type strains of the closest related species. (C) Ecological origin of the studied strains.

Whole-genome phylogenetic analysis using REALPHY placed strain B7 in a separate clade, separate from *B. stratosphericus* and *B. pumilus*, and revealed high similarity among *B. safensis* strains, which clustered into a single clade (Fig 1). The strains B7 and GX-H6 were grouped within a more inclusive clade within the *B. safensis* clade, indicating a close phylogenetic relationship. Species identification based on ANI and dDDH analyses (Fig 1) confirmed the REALPHY results and supported the classification of strain B7 as *B. safensis*. The highest ANI and dDDH values — 98.3 and 86.6%, respectively — were observed between *B. safensis* strain B7 and the type strain GX-H6. In contrast, lower values were recorded for other closely related species (Fig 1), particularly between *B. safensis* B7 and *B. pumilus* B2_10, as well as *B. stratosphericus* KcNO-8. The dDDH values in these cases were well below 70%, which is the established threshold for species delineation among bacteria [39].

### Genome annotation

**Fig 2:**
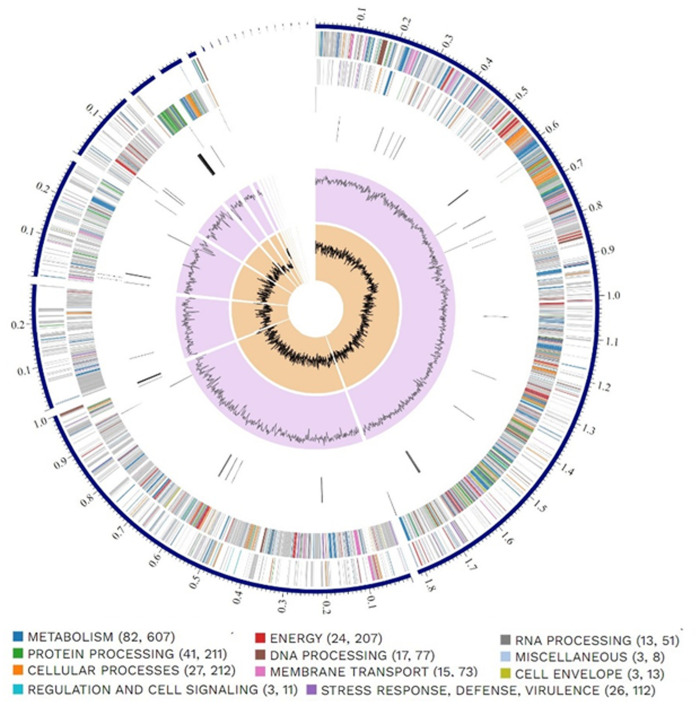
Circular visualization of bacterial genome annotations. The genome map was generated using the Bacterial and Viral Bioinformatics Resource Center (BV-BRC; https://www.bv-brc.org/). From the outermost to the innermost rings, the diagram displays assembled contigs, coding sequences (CDSs) on the forward and reverse strands, RNA-encoding genes, CDSs associated with antimicrobial resistance genes, CDSs associated with virulence factors, GC content, and GC skew. Coding sequences are color-coded according to their assigned functional subsystems.

**Fig 3:**
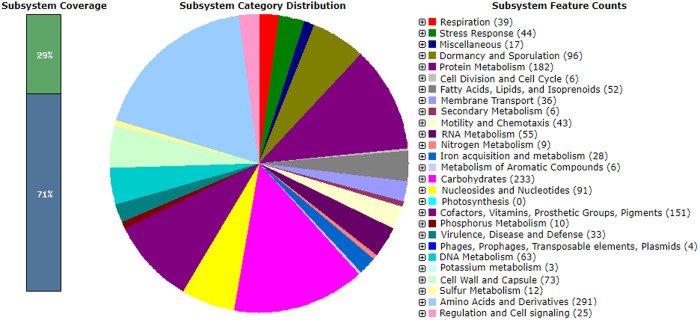
Schematic overview of subsystem coverage, subsystem category distribution, and subsystem feature counts in the annotated genome of *B. safensis* strain B7, predicted using SEED Viewer v2.0. The graphic was generated using RAST SEED Viewer v.2.0. Genomic features are color-coded according to their functional classification.

Annotation of the bacterial genome via the PATRIC platform (Fig 2) identified a total of 3,878 protein-coding sequences (CDS), alongside 74 tRNA genes, 9 rRNA genes, and a single transfer-messenger RNA (tmRNA). Additionally, 36 genes were annotated as known homologs related to antibiotic resistance, drug targets, transport mechanisms, and virulence factors (S1 Table). Complementary analysis using the RAST annotation server revealed 318 subsystems, with an overall subsystem coverage of 29% (Fig 3). The most prominent functional category was amino acid and derivative metabolism, comprising 291 genes, followed by genes involved in carbohydrate metabolism (233), protein metabolism (182), and the biosynthesis of cofactors, vitamins, prosthetic groups, and pigments (151). Further functional categories included dormancy and sporulation (96 genes), nucleoside and nucleotide metabolism (91), cell wall and capsule formation (73), DNA metabolism (63), RNA metabolism (55), fatty acid, lipid, and isoprenoid biosynthesis (52), stress response (44), motility and chemotaxis (43), respiration (39), membrane transport (36), and virulence, disease, and defense mechanisms (33). The dataset also included 28 genes associated with iron acquisition and metabolism, and 17 genes were linked to miscellaneous functions. Moreover, 25 genes were classified under regulation and cell signaling, while 4 genes were associated with mobile genetic elements, including phages, prophages, transposable elements, and plasmids. Additional functional assignments included sulfur metabolism (12 genes), phosphorus metabolism (10), and nitrogen metabolism (9). The least represented categories were secondary metabolism (6 genes), aromatic compound metabolism (6), and potassium metabolism (3).

### Genome comparison with closely related *B. safensis* strains

**Fig 4:**
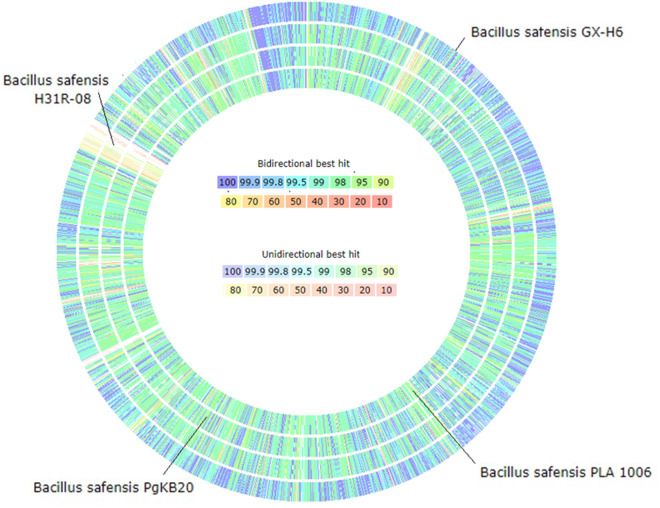
Comparison of predicted protein sequences between *B. safensis* strain B7 and closely related *B. safensis* strains. Protein sequences were compared using the sequence-based comparison tool in the RAST SEED viewer, with strain B7 set as the reference. The colour scale indicates the percentage similarity between predicted protein sequences.

The Multi-Genome Compare tool in the SEED platform revealed a high level of similarity between strains B7 and GX-H6 (Fig 4), with approximately 3,146 proteins exhibiting more than 98% sequence identity. In comparison, the number of proteins sharing >98% similarity with strain B7 was 2,231 in PgKB20, 2,209 in H31R-08, and 2,137 in PLA 1006.

**Fig 5:**
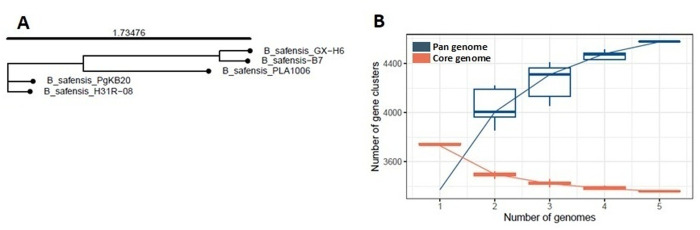
Pan-genome analysis of *B. safensis* strain B7 and four closely related *B. safensis* strains. (A) Phylogenetic tree based on whole-genome sequences. (B) Number of pan and core gene clusters across the analysed *B. safensis* genomes.

Likewise, pan-genomic phylogenetic tree analysis using the IPGA tool showed that strain B7 is most closely related to *B. safensis* strain GX-H6 (Fig 5A) which is consistent with the ANI and dDDH results (Fig 1B). Pan-genome profiling revealed that as the number of *B. safensis* genomes included in the analysis increased, the number of pan gene clusters rose sharply to 4,576, while the number of core gene clusters declined to 3,360, gradually reaching a plateau (Fig 5B). This pattern suggests that more core genes are shared across the genomes compared to the accessory or unique genes, supporting the concept of an open pan-genome in *B. safensis*. This open nature reflects genome divergence and potential adaptive strategies within the species.

**Fig 6:**
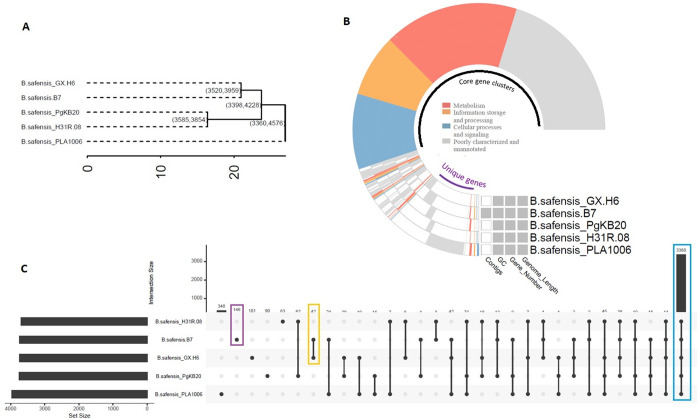
Distribution and diversity of gene clusters among *B. safensis* strains based on Pan-Genome analysis. (A) Phylogenetic tree based on Clusters of Orthologous Groups (COG). Number in parentheses indicate the number of shared gene clusters. (B) Cluster Share Analysis based on COG annotation, showing core and unique gene clusters, number of contigs and genes, GC content, and genome length. Circles represent the genomes of strain B7 and related *B. safensis* strains. Colours in the circles refer to gene function categories. The black arc and purple arc represent the core and unique gene clusters, respectively. (C) Upset plot illustrating the distribution of unique and shared genes clusters across *B. safensis* strains. Purple rectangles represent genes unique to strain B7; orange rectangles denote genes shared between B7 and its closest relative GX-H6; black rectangles indicate genes shared among all strains.

Classification of the core gene clusters based on COG annotation showed that 31.29% of core gene were involved in metabolism, 17.51% in cellular processes and signalling, and 14.62% in information storage and processing, while 36.56% were not annotated or poorly characterized (Fig 6B). Pan-genome analysis showed that the number of shared gene clusters across all strain reached 3,360, while the number of unique gene clusters per genome ranged from 63 to 348 (Fig 6C). Consistent with previous genomic phylogeny results, the COG-based phylogenetic tree confirmed that strain B7 is most closely related to *B. safensis* GX-H6, sharing approximately 3,520 gene clusters (Fig 6A). In addition, strain B7 harboured 146 novel gene clusters, representing 3.9% of its genome. These included 6 gene clusters associated with cellular processes and signalling, 10 with information storage and processing, 13 with metabolism, and 117 classified as not annotated or poorly characterized gene clusters. The analysis also revealed that strain B7 shared 42 unique genes with its closest relative, *B. safensis* GX-H6 (Fig 6C).

### Genotypic features of strain B7 involved in plant colonization and interaction

**Fig 7:**
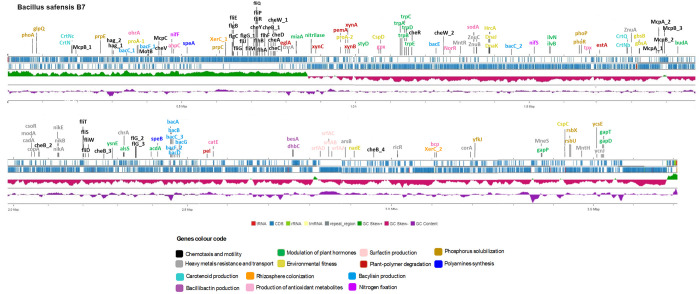
Genome map of *Bacillus safensis* strain B7 generated through Proksee. The outer lines (blue) show the annotation, location, and direction of expression of predicted genes, the middle line indicates the GC skew [(G–C)/(G+C)], with positive values in green and negative values in violet red. The inner line (purple) indicates the % GC content. Genes of interest related to beneficial plant–bacteria interactions are highlighted, with colours assigned according to their function.

Genome annotation of strain B7 revealed the presence of genes associated with the production of phytohormones, siderophores, antibiotics, lytic enzymes, and polyamines, as well as genes involved in nutrient provision and heavy metal resistance (S2 Table, Fig 7). These genetic traits support the strain’s ability to interact effectively with plants, promoting growth and enhancing resistance to stress.

Motility, chemotaxis, and plant-polymer degrading enzymes are critical traits for endophytic colonization, enabling bacteria to penetrate plant tissues, migrate internally, and colonize specific plant organs. The genome of strain B7 encodes 24 genes involved in motility and 18 genes in chemotaxis (S2 Table, Fig 7), which likely contribute to its efficient plant colonization. Furthermore, the genome harbours the *xerC1* and *xerC2* genes encoding site-specific tyrosine recombinases, which have been previously reported as crucial for plant colonization [39]. In addition, genes responsible for surfactin production (*srfAA*, *srfAB*, *srfAC*, and *srfAD*) were identified. Surfactin is a lipopeptide known for its antimicrobial properties and its role in plant growth promotion by enhancing root development and nutrient uptake [40].

PGPB often produce a variety of extracellular and intracellular plant-polymer lytic enzymes such as chitinases, β 1, 3- glucanases, proteases, cellulases, and lipases. The genome of strain B7 was found to encode several such cell wall-degrading enzymes, including glucanase (*eglA*), xylanases (*xynA*, *xynB*, and *xynC*), lipase (*EstA*), pectylhydrolase (*PemA*), and pectate lyase (*Pel*). These enzymes have versatile roles; they are involved in (i) gaining entry into plant tissues and assuring systemic spread into epidermis cells through the localized breakdown of plant cell walls; (ii) plant growth promotion by aiding in cell expansion; (ii) plant protection by degrading pathogen cell walls and the extracellular virulence factors and stimulating the plant immune system [41,42].

Polyamines such as spermine and putrescine—phytohormone-like natural compounds—have been shown to be crucial for successful plant rhizosphere colonization. They also promote plant growth and increase tolerance to various abiotic stresses [43]. Two genes involved in polyamine biosynthesis were identified: *speB* (agmatinase), involved in spermine production, and *speA* (arginine decarboxylase), involved in putrescine synthesis.

Plant-associated bacteria can affect plant physiology through the synthesis of compounds that modulate plant hormones —such as indole-3-acetic acid (IAA), acetoin (3-hydroxy-2-butanone), and cytokinins —or through the metabolization of compounds like phenylacetic acid (PAA), gamma- aminobutyric acid (GABA), and the stress-related ethylene precursor 1-aminocyclopropane-1-carboxylic acid (ACC) [44]. Plant-associated bacteria can promote plant growth and enhance resistance to abiotic stresses through the production of auxins, particularly IAA [45]. Genome analysis of strain B7 revealed the presence of genes involved in the L-tryptophan-dependent pathway of IAA production, including *trpA*, *trpB*, *trpC*, *trpD*, *trpE*, *trpS*, *YsnE*, and *blr3397*. Acetoin, a volatile compound produced by plant-associated bacteria, can promote plant growth by stimulating root formation, eliciting induced systemic resistance, and enhancing drought tolerance [46]. Genes involved in acetoin synthesis, encoding acetolactate synthase (*alsS*, *ilvN*, and *ilvB*) and acetolactate decarboxylase (*budA*), were identified in the genome of strain B7. Moreover, the gene *acdA*, which encodes an enzyme that cleaves (ACC), may contribute to reducing ethylene levels in plants exposed to environmental stress [46]. The *styD* gene, also present in the B7 genome, is associated with PAA production, a compound with known antimicrobial properties [47]. PAA is also endowed with ISR activation and the stimulation of root growth [48]. Strain B7 carries genes required for GABA metabolization (*gapT*, *gapD*, and *gapP*), which are known to support synergistic plant-microbe interactions, leading to enhanced plant growth, and tolerance to both biotic and abiotic stresses [49]. Furthermore, the genome of strain B7 contains the *miaA* gene, which is involved in cytokinin production. Cytokinin-producing- bacteria have been reported to increase plant growth under biotic and abiotic stresses [50,51].

PGPR are known to produce several secondary metabolites such as polyketides (PKs), bacteriocins, and lipopeptides (LPs) that play essential roles for applications in agriculture. They are vital for bacterial activities in suppressing disease pressure in plants through antimicrobial activities and activation of plant defence, and they are important for biofilm formation and root colonization of crop plants. LPs and PKs are produced by non-ribosomal peptide synthetases (NRPSs) and polyketide synthases (PKSs), respectively [52]. According to the AntiSMASH, BAGEL and NapDoS2 analyses (S3 Table), the genome of *B. safensis* B7 harbours bioactive gene clusters (BGCs) and genes encoding NRPSs (lichenysin, bacilysin, Bacillibactin, and surfactin), a PKS (T3PKS), ribosomally synthesized post-translationally modified peptides (RiPPs) (*BhlA*/*UviB* family holin-like peptide, class I and class II bacteriocins), and other secondary metabolites.

On the one hand, Phosphorus is a key mineral essential for plant growth and vitality, and beneficial bacteria possess genomic features that can facilitate the remineralization of organic phosphate compounds, making them available to plants from soils and residues [53]. Genes coding for enzymes involved in phosphate metabolism found in the genome of strain B7 include, among others, genes coding for phosphatases (*PhoA*, *YcsE*, *PrpC*, *PrpE*, *YfkJ*, *RsbX*, *RsbU*), phosphate regulons (*PhoR*, *PhoP*), and phosphodiesterases (*glpQ*). On another hand, nitrogen is also a key macronutrient essential for plant growth. Biological nitrogen fixation provides a natural means of supplying nitrogen to plants [54]. Genes involved in nitrogen fixation, including *nifS* (encoding cysteine desulfurase) and *nifF* (encoding flavodoxin), were also predicted in the genome of strain B7.

More genes related to beneficial plant–bacteria interactions are summarized in S2 Table and Fig 7 These genes are involved in bacterial environmental fitness (production of osmoprotectants, tolerance to high temperatures and cold shock conditions, and drug resistance), antioxidative response, heavy metal resistance and transport, and carotenoid biosynthesis.

### *B. safensis* enhance seed water uptake, germination, and seedling development in durum wheat

**Fig 8:**
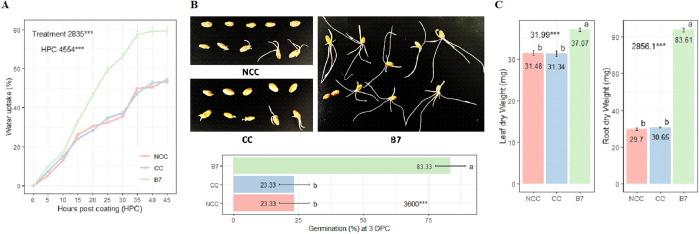
Effect of seed coating with *B. safensis* strain B7 on the kinetics of water uptake and germination of seeds, and root and shoot development. (A) Water uptake in wheat seeds. (B) Seed germination at 3 days post coating. (C) Root and shoot dry weights at 10 days post coating. Mean square values with statistical significance are shown (*** = p < 0.001). Figures were generated with R studio.

The analysis of variance showed that seed coating significantly influenced water uptake. The results of this study indicated that seed hydration was most rapid between 10 and 35 hpc for both treated and untreated seeds (Fig 8A, S1 File). In general, seed coating with *B. safensis* led to a higher rate of water uptake starting from 10 hpc, reaching 79.05% at 45 hpc, compared to the coated control, which reached 52.78% at 45 hpc. It also caused a shift in the time required to reach 50% water uptake, reducing it from 40 hours (coated control) to approximately 20 hours. Besides, ANOVA analysis showed that the seed coating significantly affected the seed germination as well as root and shoot dry weights. At 3 dpc, seed coating with *B. safensis* resulted in a fourfold increase in germination, exceeding 50% compared to the coated control (Fig 8B, S1 File). At 10 dpc, seedlings from *B. safensis*-coated seeds had higher root and shoot dry weights compared to coated controls (Fig 8C, S1 File). *B. safensis* induced a greater stimulation of root growth compared to shoot growth, illustrated by a 2.7-fold increase in root dry weight and a 1.2-fold increase in shoot dry weight.

In fact, the rate and level of seed imbibition play an important role in grain biology, particularly in germination, growth, and developmental processes, and are controlled by three main factors: seed composition, water availability in medium, and the water-permeable properties of the seed coat [55]. The water permeability of the seed is determined by the chemical composition and structural characteristics of the seed bran, also known as the seed coat [56,57], and the aleurone cell walls in the endosperm [58]. According to the literature, the seed bran of wheat is relatively thick and contains lipophilic fibres (xylans, cellulose, and lignin), which act as a barrier to water uptake [59–61]. The aleurone cell walls, primarily composed of two polymers—arabinoxylan and β-glucan—serve also as a barrier to water diffusion [58,62]. In addition, genome analysis of strain B7 identified the presence of a set of genes coding for the seed cell wall-degrading enzymes, including glucanase (*eglA*) and xylanases (*xynA*, *xynB*, and *xynC*). Indeed, it has been reported that *B. safensis* strains are capable of producing a wide range of enzymes such as amylase, cellulase, protease, lipase, xylanase, chitinase, inulinase, keratinase, and *β*-galactosidase [13]. Based on these findings, it could be assumed that following seed coating, the production of bacterial lytic enzymes might play a major role in modifying seed structure by breaking down the aforementioned barriers in the seed coat and the endosperm. This would increase water permeability, leading to greater and faster water uptake, and an enhanced germination, as observed in *B. safensis*-coated seeds.

Similarly, as reported by Lateef et al. [13], the genome of strain B7 is genetically equipped with growth-promoting traits such as phosphate solubilization, and the production of siderophore, indole-3-acetic acid, and 1--1-carboxylate deaminase, which is thought to play a major role in the observed plant growth promotion. Notably, *B. safensis* strain B7 has demonstrated great potential in promoting root development. In agreement with these results, the genome contains genes involved in root development enhancement, including those responsible for surfactin production (*srfAA*, *srfAB*, *srfAC*, and *srfAD*), acetoin synthesis (*alsS*, *ilvN*, *ilvB*, and *budA*), and phenylacetic acid production (*styD*). Chakraborty et al. [63] also reported that the application of *B. safensis* had a more pronounced impact on root growth than on shoot growth in wheat.

### *B. safensis* reduces pathogen mycelial growth and triggers systemic induced resistance (ISR) in plants

#### Antifungal activity of *B. safensis.*

**Fig 9:**
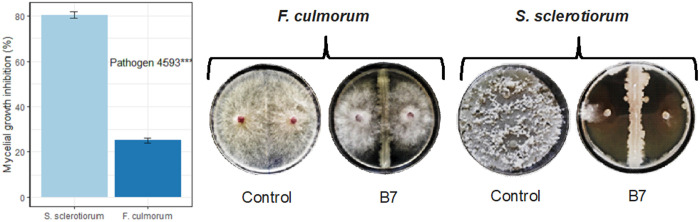
Effect of *B. safensis* strain B7 on mycelial growth of *F. culmorum and S. sclerotiorum* in a dual culture assay. Mean square values with statistical significance are shown (*** = p < 0.001). Figures were generated using R studio.

The analysis of variance showed that *B. safensis* strain B7 significantly affected the mycelial development of both pathogens. Strain B7 exhibited higher antifungal activity against *S. sclerotiorum*, with an inhibition rate of 80%, while it inhibited only 25% of *F. culmorum* mycelial growth (Fig 9, S2 File). This antifungal effect may be attributed in part to antibiotic resistance genes, drug targets genes, transporters, and virulence factor genes identified in strain B7 by PATRIC (S1 Table), as well as to antimicrobial secondary metabolites predicted by antiSMASH, including surfactin, lichensyin, bacylisin, bacillibactin, and bacteriocins (Table S3).

The differential activity of *B. safensis* against *S. sclerotiorum* and *F. culmorum* is likely due to a combination of the pathogens’ characteristics and the interaction dynamics between bacterial metabolites (lipopetides such as surfactin, bacylisin, bacillibactin, and cell wall lytic enzymes) and the fungal pathogens. *S. sclerotiorum* appears to be more vulnerable to *B. safensis* metabolites due to its simpler cell wall structure and fewer resistance mechanisms. In contrast, *Fusarium* species have cell walls that are richer in chitin, β-glucans, and α-glucans, with highly interlinked layers, and possesses enhanced structural defences through cross-linked proteins and robust β-1,6-glucans, making them more resistant to enzymatic degradation. Besides, the production of oxalic acid by *S. sclerotiorum* creates an acidic environment that may enhance the activity of *Bacillus*-produced metabolites, which they are often optimized for low pH conditions. Conversely, *F. culmorum* produces mycotoxins and biofilm, which could reduce the effectiveness of *Bacillus* antifungal compounds [64,65].

#### *B. safensis* potential in triggering the induced systemic resistance.

**Fig 10:**
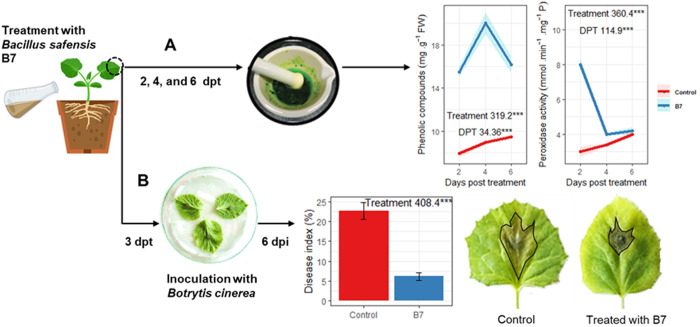
Effect of *B. safensis* B7 on melon plants. (A) Phenolic content and peroxidase activity. (B) Disease index and symptoms of gray mold caused by *B. cinerea* in melon leaves pretreated with *B. safensis* B7, compared to untreated control.

Two assays were applied in controlled conditions to determine whether treatment with *B. safensis* would induce ISR in plants and protect them from biotic stress. These assays consisted of (i) quantifying peroxidase activity and phenolic compounds levels in *B. safensis*-treated plants, as both are involved in the reactive oxygen species (ROS)-scavenging-mediated signalling within the plant (Fig 10A), and (ii) challenging *B. safensis*-treated plants with gray mold disease caused by *B. cinerea* (Fig 10B).

On the one hand, the analysis of variance showed that both treatment and time had a significant effect on the kinetics of peroxidase activity and phenolic content in melon seedlings. *B. safensis*-treated plants exhibited a peak in peroxidase activity (Fig 10A, S3 File) at 2 dpt, reaching 8 mmol.min^-1^. mg^-1^ P, while the activity in control plants continued to decrease at 4 and 6 dpt, eventually reaching the same level as in the treated plants. On the other hand, the treatment with *B. safensis* induced an overall increase in phenolic content, ranging from 15.5 to 20 mg. g^-1^ FW, over the time course, compared to control plants, which showed lower values ranging from 7.6 to 9.7 mg. g^-1^ FW (Fig 10A, S3 File).

PGPR can induce oxidative stress in plants as part of the signalling process involved in their beneficial interactions. PGPB make plants perceive them as beneficial stressors through the secretion of various metabolites, including plant cell wall-degrading enzymes, auxins, salicylic acid, VOCs (such as acetoin and 2,3-butanediol), exopolysaccharides, and siderophores [66]. Peroxidases and phenolic compounds help mitigate this stress by detoxifying ROS, maintaining cellular homeostasis, and preventing damage to cellular structures. These metabolites can directly or indirectly enhance the plant’s ability to cope with stress and strengthen its defence responses. At its core, the kinetics study of peroxidase activity and phenolic content indicates that *B. safensis* triggers a short-term stress response. This signalling suggests that *B. safensis* facilitates a systemic transmission of signals from the roots to the leaves, likely participating in broader metabolic changes within the plant.

According to the variance analysis of the detached leaf assay, the treatment significantly affected the lesion size caused by *B. cinerea*. At 6 dpi, treatment with *B. safensis* reduced the disease index of gray mold to 6.1%, compared to the control, in which the disease index reached 22.6% (Fig 10B, S3 File). These results indicate that treatment with B7 stimulated the defence mechanisms in melon plants when challenged with the pathogen. This effect could be attributed to the induction of ISR. In other words, prior exposure of the roots to *B. safensis* strain B7 established a primed state in the leaves, which displayed a faster and/or stronger activation of ISR upon *B. cinerea* infection.

### *B. safensis* improves the yield components of durum wheat under two different bioclimates and water regimes

#### Variation in crop yield related to contrasting environmental conditions.

The two cropping seasons 2016–2017 and 2019–2020, differed in rainfall amount and distribution, applied water regimes, and temperatures (Tables 1 and 2). During the 2016–2017 season, plants were supplemented with two different irrigation levels to meet 100% and 50% of the crop water requirement. In contrast, in the 2019–2020 season, plants were grown under rainfed conditions, and the total rainfall received was lower than the water amounts supplied to plants during the 2016–2017 season. These contrasting environmental conditions led to significant differences in crop yield. The lower rainfall under rainfed conditions during the 2019–2020 season undoubtably resulted in reduced crop yields compared to those observed in 2016–2017 under controlled irrigation.

#### Field Experiments in the semi-arid and arid regions of Tunisia.

**Fig 11:**
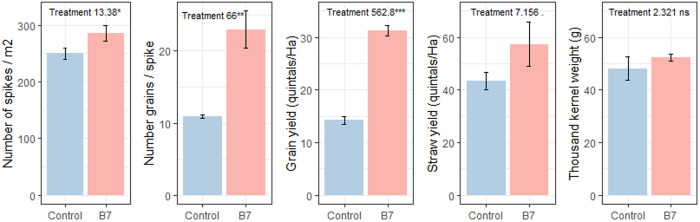
Effect of treatments and supplemental irrigation on agronomic components of durum wheat in the 2019–2020 crop season at the experimental station of Mateur (semi-arid region of Tunisia). Mean square values with statistical significance are shown (ns: non-significant;. = p < 0.1; * = p < 0.05; ** = p < 0.01; *** = p < 0.001).

Under the rainfed regime in the semi-arid region of Tunisia (cropping season 2019–2020), seed coating with *B. safensis* resulted in a higher number of spikes per m^2^ (Fig 11, S4 File), as well as increased numbers of grains per spike, grain yield, straw yield, and thousand kernel weight (TKW), compared to the coated control. The impact of *B. safensis* was particularly notable in the of number of grains/spike and grain yield; both of which showed increases exceeding 50%. The grain yield reached 31.3 quintals/ha.

**Fig 12:**
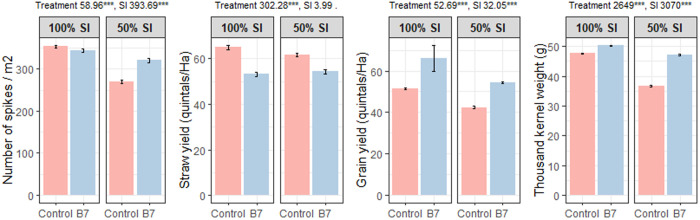
Effect of treatments and supplemental irrigation on agronomic components of durum wheat in the 2016–2017 crop season at the experimental station in Chébika (arid region of Tunisia). Mean square values with statistical significance are shown (ns: non-significant;. = p < 0.1; * = p < 0.05; *** = p < 0.001).

In the arid region of Tunisia (cropping season 2016–2017), an imposed increase in water stress severity —from 100% to 50% SI —in plants derived from coated control seeds of the “Karim” cultivar led to decreases in grain yield and all other agronomic components (Fig 12, S5 File). Previous studies have reported similar reductions in effective spikes, kernels per spike, kernel weight, and grain yield under drought stress [67].

Under moderate water stress (100% SI), and compared to coated control, seed coating with *B. safensis* resulted in a lower number of spikes per m^2^ and lower straw yield, but higher grain yield and TKW. Under severe water stress conditions (50% SI), compared to the coated control, *B. safensis*-coated seeds produced plants with a lower straw yield but higher number of spikes per m^2^, grain yield, and TKW. Notably, the grain yield of plants derived from *B. safensis*-coated seeds under severe water stress (50% SI) was higher than that of coated control plants under moderate stress (100% SI).

The potential of *B. safensis* to mitigate the effects of drought on agronomic performance may be attributed to its genome, which contains genes involved in plant growth promotion and abiotic stress tolerance. These include genes involved in the production of acetoin ((*alsS*, *ilvN*, *ilvB*, and *budA*), polyamines (*speA* and *speB*), enzymatic antioxidants (*sodA*, *KatE*, and *gpx*), non-enzymatic antioxidants (*NorR*, *OhrA*, *ahpC*, *tpx*, and *bcp*), cytokinin (*miaA*), IAA (*trpA*, *trpB*, *trpC*, *trpD*, *trpE*, *trpS*, *YsnE*, and *blr3397*), and in GABA metabolization (*gapT*, *gapD*, and *gapP*), and ACC deamination (*acdA*). Our results are consistent with the findings of Chakraborty et al. [68], who reported that *B. safensis* enhanced growth in six wheat varieties and improved their ability to withstand water stress.

## Conclusion

In this work, it was possible to confirm that seed coating with *B. safensis* strain B7 exhibited multiple growth-promoting related features, including enhanced seed germination and seedling development, improved defence system against pathogens, and increased wheat productivity under drought stress. The presence of genes in the genome of *B. safensis* strain B7 —known to play key roles in mitigating drought stress and biotic stress, as well as increasing plant productivity —supports its beneficial potential. These genes are involved in plant colonization, phytohormone synthesis, and various stress response mechanisms. However, validating the actual production of these compounds is essential for fully harnessing the potential of strain B7. Its genotypic traits may be attributed to its origin in a hostile environment, which likely shaped its adaptive capabilities. Overall, these findings, together with the complete genome of strain B7, not only contribute to our understanding of plant – microbe interactions but also provide a solid foundation for the straightforward application of this bacterium as a microbial seed coating to improve crop resilience to drought.

## Supporting information

S1 Table Antimicrobial resistance genes detected in *Bacillus safensis* strain B7. (DOCX)

S2 Table Genes predicted in *Bacillus safensis* B7 genome to be involved in plant growth-promotion activity. (DOCX)

S3 Table Summary of biosynthetic gene clusters and ribosomally synthesized and post-translationally modified peptides (RiPPs) detected in the *B. safensis* B7 genome using antiSMASH, NaPDoS2, and BAGEL4. (DOCX)

S1 Fig Optimization of the dose of Agritan® SC for wheat seed coating, based on germination percentage at 3 days post coating. Mean square values with statistical significance are shown (*** = p < 0.001). Figures were generated with R studio.
(DOCX)

S0 File Data of germination percentage used to optimize the dose of Agritan® SC for wheat seed coating. (XLSX)

S1 File Data of water uptake kinetics, seed germination, and root and shoot development. (XLSX)

S2 File Data of mycelium growth inhibition of *F. culmorum* and *S. sclerotiorum* in the dual culture assay. (XLSX)

S3 File Data of phenolic content, peroxidases activity, and gray mold disease index in *B. cinerea*-infected melon plants. (XLSX)

S4 File Agronomic data for durum wheat in the 2019–2020 cropping season at the experimental station of Mateur (semi-arid region of Tunisia). (XLSX)

S5 File Agronimc data for durum wheat in the 2016–2017 cropping season at the experimental station in Chébika (arid region of Tunisia). (XLSX)
