# Supplementary material for: Genome-driven insights into Bacillus safensis strain B7 as a seed coating agent for plant growth promotion and alleviation of biotic and abiotic stresses
Source: PLoS One. 2025 Aug 18;20(8):e0329619. doi: 10.1371/journal.pone.0329619 (PMC12360542; doi:10.1371/journal.pone.0329619)
Supplement: S1 Table — (DOCX) [file pone.0329619.s001.docx]

**Table S1. Antimicrobial resistance genes detected in *Bacillus safensis* strain B7**

| Antimicrobial Resistance Mechanism | Genes |
| --- | --- |
| Antibiotic inactivation enzyme | *CatA6* family |
| Antibiotic target in susceptible species | *Alr*, *Ddl*, *dxr*, *EF-G*, *EF-Tu*, *folA*, *Dfr*, *folP*, *gyrA*, *gyrB*, *inhA*, *fabI*, *Iso-tRNA*, *kasA*, *MurA*, *rho*, *rpoB*, *rpoC*, *S10p*, *S12p* |
| Antibiotic target protection protein | *BcrC* |
| Antibiotic target replacement protein | *fabL* |
| Efflux pump conferring antibiotic resistance | *BceA*, *BceB*, *EbrA*, *EbrB* |
| Gene conferring resistance via absence | *gidB* |
| Protein altering cell wall charge conferring antibiotic resistance | *GdpD*, *MprF*, *PgsA* |
| Regulator modulating expression of antibiotic resistance genes | *BceR*, *BceS*, *LiaF*, *LiaR*, *LiaS* |
