# Supplementary material for: Genome-driven insights into Bacillus safensis strain B7 as a seed coating agent for plant growth promotion and alleviation of biotic and abiotic stresses
Source: PLoS One. 2025 Aug 18;20(8):e0329619. doi: 10.1371/journal.pone.0329619 (PMC12360542; doi:10.1371/journal.pone.0329619)
Supplement: S2 Table — (DOCX) [file pone.0329619.s002.docx]

**Table S2. Genes predicted in *Bacillus safensis* B7 genome to be involved in plant growth-promotion activity**

| Pathway | | Genes | Product |
| --- | --- | --- | --- |
| Surfactin production | | *srfAA*, *srfAB*, *srfAC*, *srfAD* | Surfactin synthase |
| Bacillibactin poduction | | *DhbC* | Isochorismate synthase |
|  |  | *BesA* | Ferri-bacillibactin esterase |
| Bacylisin production | | *BacA*, *BacB*, *BacC1*, *BacC2*, *BacC3*, *BacE*, *BacF1*, *BacF2*, *BacG* | Bacylisin |
| Rhizosphere colonization | | *XerC1*, *XerC2* | Tyrosine recombinase |
| Motility | | *FlhA*, *FlhB*, *FlhF*, *Hag1*, *Hag2*, *FlgB*, *FlgC*, *FlgG1*, *FlgG2*, *FlgG3*, *FliD*, *FliE*, *FliG*, *FliJ*, *FliM*, *FliQ*, *FliP*, *FliR*, *FliT*, *FliS*, *FliW*, *MotB* | Flagellin biosynthesis and regulation |
|  |  | *SwrD*, *SwrC* | Swarming motility protein |
| Chemotaxis | | *CheA*, *CheB1*, *CheB2*, *CheB3*, *CheB4*, *CheC*, *CheD*, *CheR*, *CheW1*, *CheW2*, *CheV*, *CheY*, | Chemoreceptors |
|  |  | *McpA1*, *McpA2*, *McpB1*, *McpB2*, *McpB3*, *McpC* | Methyl-accepting chemotaxis proteins |
| Plant-polymer degradation | Glucanases | *eglA* | Endoglucanase A |
|  | Xylanase | *xynA* | Glycosyl hydrolase |
|  |  | *xynB* | Endo-1,4-beta-xylanase |
|  |  | *xynC* | Glucuronoxylanase |
|  | Lipase | *EstA* | GDSL-type lipase |
|  | Pectin degradation | *PemA* | Pectin pectylhydrolase A |
|  |  | *pel* | Pectate lyase |
| Polyamines synthesis | | *speA*, *speB* | Putrescine/ spermidine |
| Carotenoid production | | *CrtN*, *CrtNc*, *CrtNb*, *CrtQ*, | Phytoene desaturase |
| Modulation of plant hormones | Acetoin synthesis | *alsS*, *ilvN*, *ilvB* | Acetolactate synthase |
|  |  | *budA* | Alpha-acetolactate decarboxylase |
|  | ACC deamination | *acdA* | Acyl-coa dehydrogenase |
|  | L-tryptophan Production; IAA  Production | *trpA*, *trpB* | Tryptophan synthase |
|  |  | *trpC* | Indole-3-glycerol phosphate synthase |
|  |  | *trpD* | Anthranilate phosphoribosyltransferase |
|  |  | *trpE* | Anthranilate synthase component I |
|  |  | *trpS* | Tryptophan--trna ligase |
|  |  | *YsnE* | N-acetyltransferase |
|  |  | *blr3397* | Nitrilase |
|  | Phenyl acetic acid (PAA) metabolization | *styD* | Phenylacetaldehyde dehydrogenase |
|  | Gamma-aminobutyrate (GABA) metabolization | *gabT* | 4-aminobutyrate aminotransferase |
|  |  | *gapD* | Succinate-semialdehyde dehydrogenase |
|  |  | *gapP* | GABA permease |
|  | Cytokinin (Zeatin) biosynthesis and  Transformation | *MiaA* | Trna (adenosine(37)-N6)-dimethylallyltransferase |
| Phosphorus solubilization | | *PhoA* | Alkaline phosphatase |
|  |  | *YcsE* | Phosphoglycolate phosphatase |
|  |  | *PrpC* | Protein phosphatase |
|  |  | *prpE* | Bis(59-nucleosyl)-tetraphosphatase |
|  |  | *yfkJ* | Tyrosine-phosphatase |
|  |  | *RsbX*, *RsbU* | Phosphoserine phosphatase |
|  |  | *PhoR*, *PhoP* | Phosphate regulon |
|  |  | *glpQ* | Glycerophosphodiester phosphodiesterase |
| Nitrogen fixation | | *nifS* | Cysteine desulfurase |
|  |  | *nifF* | Flavodoxin |
| Heavy metals resistance and transport | | *NikA*, *NikB*, *NikE*, *CnrA* | Nickel |
|  |  | *RicR*, *YcnJ*, *copA*, *CsoR* | Copper |
|  |  | *CnrA*, *CorA* | Cobalt |
|  |  | *ZnuA*, *ZnuB*, *ZnuC*, *cadA* | Zinc |
|  |  | *ModA* | Molybdenum |
|  |  | *MntR*, *MntH*, *MneS* | Manganese |
|  |  | *cadA* | Cadmium |
|  |  | *chrA* | Chromate |
|  |  | *arsB* | Arsenic |
| Production of antioxidant metabolites | | *sodA* | Superoxide dismutase |
|  |  | *gpx* | Glutathione peroxidase |
|  |  | *catE* | Catalase |
|  |  | *OhrA* | Organic hydroperoxide resistance protein |
|  |  | *NorR* | Nitric oxide reductase transcription regulator |
|  |  | *tpx* , *ahpC*, *bcp* | Peroxiredoxins |
| Environmental fitness | | *DnaJ*, *DnaK*, *HrcA*, | Heat shock protein synthesis and regulation |
|  |  | *nadE* | NAD-synthetase |
|  |  | *CspC*, *CspD* | Cold shock protein synthesis |
|  |  | *gbsA*, *gbsB* | Glycine betaine |
|  |  | *proA1*, *proA2* | Proline |
