# Supplementary material for: Genome-driven insights into Bacillus safensis strain B7 as a seed coating agent for plant growth promotion and alleviation of biotic and abiotic stresses
Source: PLoS One. 2025 Aug 18;20(8):e0329619. doi: 10.1371/journal.pone.0329619 (PMC12360542; doi:10.1371/journal.pone.0329619)
Supplement: S3 Table — (DOCX) [file pone.0329619.s003.docx]

**Table S3. Summary of biosynthetic gene clusters and ribosomally synthesized and post-translationally modified peptides (RiPPs) detected in the *B. safensis* B7 genome using antiSMASH, NaPDoS2, and BAGEL4**

| **antiSMASH** | **Type of cluster** | **Predicted cluster with BlastP** | | | **Start - End** | **Similarity (%)** |
| --- | --- | --- | --- | --- | --- | --- |
|  | NRP-metallophore, NRPS | Bacillibactin | | | 922,573 - 974,301 | 100% |
|  | NRPS | Lichenysin | | | 1 - 73,032 | 100% |
|  | NRPS | Bacilysin | | | 626,353 - 667,774 | 100% |
|  | RRE-containing | PqqD Pyrroloquinoline quinone chaperon protein | | | 9,661 - 30,566 | 100% |
|  | Terpene, NI-siderophore | Phytoene/squalene synthase, Iuca/Iucc siderophore | | | 175,491 - 213,338 | 100% |
|  | Betalactone | - | | | 914,440 - 942,849 | - |
|  | Terpene | Prenyltransferase/squalene oxidase | | | 999,889 - 1,021,763 | - |
|  | T3PKS | Type III polyketide synthase | | | 1,061,193 - 1,102,287 | - |
|  | Betalactone | - | | | 1,546,385 - 1,578,639 | - |
| **NaPDoS2** | **Type of cluster** | **Predicted cluster** | | **Domain class** | **Start - End** | **Similarity** (%) |
|  | NRPS | Bacillibactin | | LCL | 950315 - 951646 | 60 |
|  |  |  |  | Starter | 947183 - 948481 | 56 |
|  | NRPS | Lichenysin | | DCL | 31515 - 30220 | 60 |
|  |  |  |  | LCL | 13262 - 11958 | 56 |
|  |  |  |  | Starter | 53011 - 51710 | 50 |
|  | NRPS | Surfactin | | DCL | 42265 - 40958 | 58 |
|  |  |  |  | Epimerization | 20529 - 19228 | 53 |
|  | KS | Bacillus subtilis FAS | | Type II FAS | 234286 - 235524 | 84 |
| **BAGEL4** | **Class of RiPPs** | | **Predicted RiPPs with BlastP** | | **Start - End** | **Similarity (%)** |
|  | Class I bacteriocins (Sactipeptide) | | Radical *SAM*/*CxCxxxxC* motif protein *YfkAB* | | 78914 - 98914 | 100% |
|  | *Uvib* family holin-like peptides | | *BhlA*/*UviB* family holin-like peptide | | 232279 - 252468 | 100% |
|  | Class II bacteriocins (closticin_574) | | - | | 587240 -608119 | - |

KS: ketide synthase; NRP: non-ribosomal peptide; *NRPs* nonribosomal peptide synthetases, *PKS* polyketide synthase, RRE*:* *RiPP recognition element*, FAS: Fatty acid synthase, RiPPs: Ribosomally synthesized and post-translationally modified peptides, NI: NRPS-independent
