# Supplementary material for: Genome-driven insights into Bacillus safensis strain B7 as a seed coating agent for plant growth promotion and alleviation of biotic and abiotic stresses
Source: PLoS One. 2025 Aug 18;20(8):e0329619. doi: 10.1371/journal.pone.0329619 (PMC12360542; doi:10.1371/journal.pone.0329619)
Supplement: S1 Fig — Mean square values with statistical significance are shown (*** = p < 0.001). Figures were generated with R studio. (DOCX) [file pone.0329619.s004.docx]

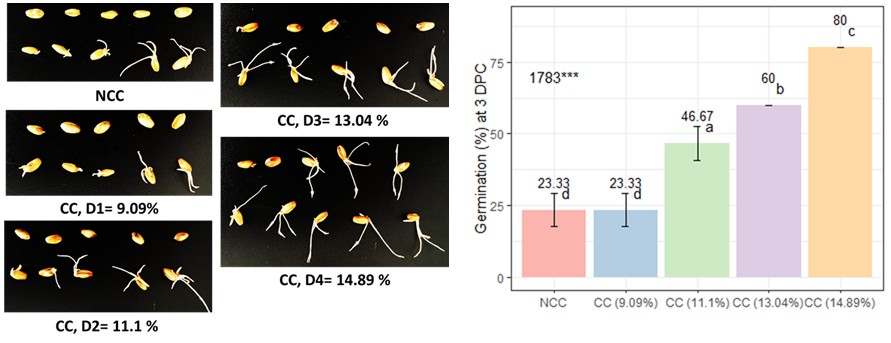


**Figure S1.** **Optimization of the dose of Agritan® SC for wheat seed coating, based on germination percentage at 3 days post coating.** Mean square values with statistical significance are shown (***= p < 0.001). Figures were generated with R studio
